# Inventing Engineered Organoids for end-stage liver failure patients

**DOI:** 10.1007/s10735-022-10085-7

**Published:** 2022-07-27

**Authors:** Radiana D Antarianto, Amer Mahmood, Angela Giselvania, Ayu AA Prima Asri Dewi, Jatmiko Gustinanda, Jeanne Adiwinata Pawitan

**Affiliations:** 1 Department of Histology Fakultas Kedokteran Universitas Indonesia, Jakarta Pusat, Indonesia; 2 Stem cell and tissue engineering research cluster IMERI UI, Jakarta, Indonesia; 3 Stem Cell Unit, Department of Anatomy, King Saud University, Riyadh, Saudi Arabia; 4 Department of Radiotherapy RS Cipto Mangunkusumo, Jakarta, Indonesia; 5 Doctoral Program in Biomedical Science Fakultas Kedokteran Universitas Indonesia, Jakarta Pusat, Indonesia; 6 Department of Histology, Fakultas Kedokteran dan Ilmu Kesehatan Universitas Warmadewa, Bali, Indonesia; 7 Master Program in Biomedical Science Fakultas Kedokteran Universitas Indonesia, Jakarta Pusat, Indonesia; 8 Undergraduate Medicine Program Fakultas Kedokteran Universitas Indonesia, Jakarta Pusat, Indonesia; 9 Integrated Service Unit Stem Cells RS Cipto Mangunkusumo, Jakarta, Indonesia

**Keywords:** ESLD, Liver transplant, Artificial liver, Liver organoid, Bioartificial liver

## Abstract

End-stage liver disease (ESLD) is a term used clinically in reference to a group of liver diseases with liver transplantation as the choice of treatment. Due to the limitations of liver transplantation, alternative treatments are needed. The use of primary human hepatocytes represents a valid alternative treatment, but the limitations related to hepatocyte quality, viability, function, conservation, and storage need to be overcome. Transplanted hepatocytes have only been followed for 6–9 months. Therefore, long-term causes of failures are not yet established, including rejection, apoptosis, or other causes. Other alternative therapies to replace liver transplantation include plasmapheresis, hemodiafiltration, and artificial livers. Unfortunately, these methods are highly limited due to availability, high cost, anaphylaxis reaction, development-deposition of immune-complexes, and restricted functionality. Liver organoids, which utilize stem cells instead of ‘impractical’ adult hepatocytes, may be a solution for the development of a complex bioartificial liver. Recent studies have explored the benefits of differentiating mature hepatocytes from stem cells inside a bioreactor. When the use of human-induced Hepatocytes (hiHeps) was investigated in mouse and pig models of liver failure, liver failure markers were decreased, hepatocyte function indicated by albumin synthesis improved, and survival time increased. Bioartificial liver treatment may decrease the infiltration of inflammatory cells into liver tissue by down-regulating pro-inflammatory cytokines.

## End-stage liver disease

End-stage liver disease (ESLD) is a term clinically used in reference to a group of liver diseases with characteristics of liver microenvironment destruction. The destruction is characterized by diffuse focal necrosis of hepatocytes with advanced fibrous septa, constricted blood vessels, and blocked biliary tree structures. ESLD is a chronic condition caused by chronic inflammation, which leads to fibrosis in liver tissue. The fibrosis disrupts liver structures and functions. Liver diseases in the ESLD category include non-alcoholic fatty liver diseases and non-alcoholic steatohepatitis (NASH), alcohol-induced hepatitis-related-cirrhosis, viral hepatitis-related cirrhosis, liver cancer, inherited and metabolic diseases, and acute liver failure. The worldwide incidence and prevalence of ESLD reached the epidemic burden of 50 million patients in 2017. According to the US American Liver Foundation, from 1998 to 2007, the incidence of acute liver failure was up to 2000 patients per year. Mortality related to ESLD included 1–2 million deaths annually in Europe in 2018. Yagi et al. 2009; Ogoke et al. 2017; Verstegen et al. 2019; Bizzaro et al. 2019)

Standard therapy for ESLD is liver transplantation. 80% of ESLD patients die due to the limited availability of liver transplantation. Up to 30,000 liver transplantations are performed per year in 500 transplantation centers globally. This number only fulfills 10% of the global liver transplant (LT) need. (Giwa et al. 2017; Verstegen et al. 2019) This review discusses progress in the field of liver transplantation, bioengineering strategies to enhance pre-transplant liver graft viability, and the regenerative approach of engineering stem cell-based bioartificial livers.

## Mainstream therapy for ESLD: liver transplant limitations and room for improvement

The widely practiced standard curative therapy for ESLD is LT. Symptomatic and palliative therapy for ESLD are also used. (Potosek et al. 2014) Liver transplantation requires a surgical procedure to replace the diseased liver with a donated liver, in its entirety or in part. In Asia, living donor liver transplant (LDLT) is more frequently practiced because of cultural and religious views regarding deceased organ donation or brain death organ harvest.(Black et al. 2018) Prior to the availability of LDLT, recently deceased donor liver transplant (DDLT) was standard practice. In fact, 82–85% of donor transplants in the international registry are from deceased donors, while the rest are attained from living donors. (Black et al. 2018; Rai 2019; Manzarbeitia 2022) In DDLT, the liver from a donor can be transplanted whole (whole organ transplant) or can be split to supply multiple recipients. The split type transplant is performed by dividing a donated liver into right and left lobes, which can be used for two patients. Right lobes are usually for adult patients and the smaller left lobes are for pediatric patients. (Rai 2019; Manzarbeitia 2022)

Based on the anatomic location of the liver graft, transplantations can be divided into orthotopic or heterotopic transplants. Orthotopic liver transplantations (OLT) are performed by transplanting a donated liver into the correct anatomical position. (Mereilles et al. 2015; Manzarbeitia 2022) In 2015, over 7000 OLTs were performed. The most common complications following OLT were pulmonary, bleeding, and infectious. (Bhutiani et al. 2018) Heterotopic liver transplantations are done by placing the donated liver in an extrahepatic location, usually at the root of the mesentery. (Mereilles et al. 2015; Manzarbeitia 2022)

The establishment of LDLT for ESLD curative therapy has a long journey. In 1955, Welch described liver transplantation for the first time as a therapy for the management of liver disease. In 1958, Francis Moore performed orthotopic liver transplantation for the first time in a dog. (Chan and Fan Sheung 2008) The first human liver transplantation was performed by Thomas E. Starzl on March 1st, 1963 at the University of Colorado, to treat a 3-year-old child suffering from biliary atresia. (Vacanti and Kulig 2014; Starzl 2015; Manzarbeitia 2022) Many problems and complications were encountered, and the patient died during the procedure due to a coagulation disorder and uncontrolled bleeding. In the next four adult patients undergoing the procedure, postoperative complications ultimately led to death within 23 days after the procedure. Ischemia-reperfusion injury and rejection eventually led to liver failure or sepsis. These discouraging results caused the program to be suspended for several years. The procedure was re-initiated in 1967, stimulated by the introduction of anti-thymocyte globulin by Calne. Starzl successfully performed several liver transplantations at the University of Colorado (Meirelles Júnior et al. 2015) out of 8 children, 4 survived past the first year and 1 patient survived 3 decades. (Vacanti and Kulig 2014; Starzl 2015) The main cause of failure was sepsis, which was related to the lack of good immunosuppressive drugs.

The problem of immunosuppressive drug availability was solved by the discovery of cyclosporine. In 1979, Calne used cyclosporine for the first time in patients undergoing liver transplantation. In 1983, the National Institutes of Health approved the use of liver transplantation as a treatment for end-stage liver diseases after evaluating approximately 500 cases. In 1989, Starzl et al. reported on 1,179 patients undergoing liver transplantation with a 73% and 64% 1- and 5-year survival rate, respectively. In 1990, Starzl et al. reported the use of tacrolimus, a new immunosuppressant agent, in patients undergoing liver transplantation, who suffered rejection using conventional immunosuppressive treatment. (Meirelles Júnior et al. 2015; Starzl 2015)

The idea of using LDLT was proposed as early as 1969; however, the first attempt, made by Raia et al., was not until December 1988. The first successful LDLT was achieved by Strong et al. in Australia in 1989. The Chicago group led by Broelsch developed the first adult-to-child LDLT program, and some small series of LDLT cases were reported in the US and Europe. (Chan and Fan Sheung 2008; Vacanti and Kulig 2014)

For adult-to-adult LDLT (ALDLT), the left liver was used initially, as reported by the Shinshu group from Japan. The left lobe use for adults was often handicapped by inadequate graft size. In 1993, the Kyoto group used the right lobe in an adult-to-child LDLT for a 9-year-old recipient. The intention, in this particular case, was to avoid the precarious arterial anatomy of the donor’s left lobe. The first successful case of right lobe ALDLT was performed at Queen Mary Hospital at the University of Hong Kong in 1996. (Chan and Fan Sheung 2008) (Table 1)

**Table 1:** Liver transplantation research milestone. (modified from Chan and Fan Sheung 2008)

Investigator	Milestone	Year
Starzl	First DDLT in human	1963
Starzl	1 year survival DDLT recipients	1968
Calne	Cyclosporin A	1979
Bismuth	Adult-to-child reduced size DDLT	1984
Pichlmayr	Split graft DDLT for two recipients	1988
Raia	LDLT	1989
Strong	Adult-to-child LDLT	1990
Yamaoka	Right liver graft from adult-to-child	1994

Right hepatectomy is one of the riskiest major surgeries in living donors. Subjecting a donor, with no medical indication for surgery, to a major surgical operation with the attendant risks is an ethical challenge. The reported overall complication rate for donors is around 20%, but complication rates as high as 67% have been reported. Complication rates among different centers and complication types vary. The most common complications are wound infection, ileus, and bile leakage. In experienced centers, donor morbidity is lower than 20%. The majority of complications are Grade I and wound infections occur the most. With careful attention to biliary anatomy and guidance from intraoperative cholangiography, biliary complications are avoidable. (Chan and Fan Sheung 2008)

Donor mortality is one of the highest concerns in the community. Donor right hepatectomy carries a 0.5% donor mortality rate. The causes of donor mortality vary. In a widely publicized case, a male donor in New York succumbed to gas gangrene caused by *Clostridium perfringens* 3 days after a right hepatectomy. In Japan, a woman with hypertension died from liver failure after right liver donation; her residual left liver had non-alcoholic steatohepatitis affecting 28% of the total liver volume. Fatal pulmonary embolism also occurred in a left liver donor. A donor mother with a history of substance abuse also died from drug overdose 2 months after donation to her 3-year-old son. Based on the 0.5% donor mortality rate, it takes one donor life per 160 recipients to achieve a five-year recipient survival of 80%. Less tangible are changes in the quality of the donor’s life after donation compared to pre-donation. The long-term biological consequences of donor hepatectomy are not fully known. Nevertheless, there are demonstrable drops in white cell count and platelet counts and elevation of liver transaminases even two years after right liver donation. Quantification of quality of life changes is required for defining the strength of LDLT. (Chan and Fan Sheung 2008) Ethically, accepting higher risk for the donor simply because of the improvement of recipient outcome is not appropriate.

Fifty years after the first liver transplantation, more than 10,000 liver transplantations have been performed around the world with a high success rate (80–90%) and few rejection problems, post-operation complications, or intra-operation problems. However, for ESLD, the problem is not solved. The number of patients with ESLD is increasing faster than donor availability. Thus, the waitlist for liver transplantation to treat ESLD is long and increasing every year. (Neuberger 2004; Vacanti and Kulig 2014; Black et al. 2018)

Liver transplantation using recently deceased donors or living donors are still problematic. We can shorten the waiting time for transplantation by using LDTD and we can perform full evaluations of living donors. However, the risk of mortality and morbidity of the donor remains high. Meanwhile, the recipient obtains the whole organ in DDLT, but the operation must be performed as soon as possible, while the cadaver is available, and the patient must be medically and mentally ready. Another problem, especially in Asia, is the primitive organ donation system. The system relies on family members, thus limiting liver availability. Limitations on liver availability impose longer wait times for patients on the transplantation waitlist. In addition to the transplantation waitlist, drop out cases are still high due to ethical problems with transplantations or exclusion from the transplant waitlist due to ESLD progression to mortality. A significant percentage of patients die while waiting for a liver transplantation. (Black et al. 2018)

Ideally, if medical treatment is effective and donor availability is balanced with demand, liver transplantations could be performed with minimal waiting periods for ESLD. However, the LT problem remains complex and unresolved. A new strategy is the usage of xenotransplantation. However, this strategy raises new problems due to the high risks of acute rejection and ethical issues. (Vacanti and Kulig 2014; Black et al. 2018) Therefore, alternative therapies are needed.

## Alternative therapy for ESLD: hepatocyte transplantation

Cell therapy is an alternative to liver transplantation. Cell-based therapies include gene therapy, cell transplantation, bioartificial liver devices, and bioengineered livers. Transplantation of hepatocytes is accomplished by isolating cells from a donor’s liver and infusing the cells into a recipient. The goal of hepatocyte transplantation is to alleviate the patient’s disease progression, thus improving the survival rate while waiting for liver donation. (Vacanti and Kulig 2014; Cardoso et al. 2018)

Transplanted hepatocytes are thought to be more beneficial compared to surgery because they are less invasive and can be transplanted into multiple recipients. In addition, use of immunosuppressive agents can be minimized by this method. Another advantage of cell transplantation is the low technical difficulty. Hepatocytes can be transplanted using an intravascular catheter as opposed to the complex surgery for liver transplantation. Hepatocytes can be cryopreserved; thus, transplantation can be performed sequentially and promptly in response to the patient’s condition. Finally, the cost of this procedure is lower than the cost of surgery for liver transplantation. (Habka et al. 2015; Cardoso et al. 2018)

Primary hepatocytes, isolated from human liver, are a good source for hepatocyte transplantation, but the usage is limited due to technical problems. Hepatocytes cannot survive for long periods in vitro due to low proliferation, dedifferentiation, and susceptibility to apoptosis during the freeze-thaw procedure. An insufficient number of viable primary hepatocytes can be isolated from a donated liver. Moreover, the expansion of primary hepatocytes for transplantation is not yet possible, while maintaining hepatocyte quality, viability, function, conservation, and storage after hepatocyte isolation. Transplanted hepatocytes only survive for 6–9 months; thus, long-term causes of failures have not been established. (Vacanti and Kulig 2014; Forbes et al. 2015; Cardoso et al. 2018)

As regenerative medicine progresses, the focus of cell therapy has moved from primary hepatocytes to hepatocyte progenitor cells. Hepatocyte progenitor cells are derived from stem cells and may be able to restore the normal structure and function of the liver. Stem cells can differentiate and self-renew, making them a good alternative to reduce the limitations of hepatocyte availability. The use of stem cell-derived hepatocyte progenitor cells is a potential alternative to liver transplantation for treating ESLD. Stem cells are available from different sources, each with advantages and disadvantages. (Potosek et al. 2014; Forbes et al. 2015; Nicolas et al. 2016) Sources of stem cells include certain organs and induced pluripotent stem cells (iPSCs). (Nicolas et al. 2016; Giwa et al. 2017)

Even if hepatocytes are replaced by stem cell technology (Nicolas et al. 2016), cell transplantation still has problems.(Forbes et al. 2015) Transplanted hepatocytes delivered into the spleen or portal vessels are trapped in the proximal liver sinusoids (up to 70% of transplanted cells) and almost all are destroyed by Kupffer cells as part of their phagocytic response. Although the remaining cells could translocate from the sinusoid into the liver parenchyma, only 0.5% of the transplanted hepatocytes engraft into the recipient’s liver, and only a few of those survive and proliferate. (Attia Atta 2013; Forbes et al. 2015) Initially, the liver itself was thought to provide an ideal environment for implanted hepatocytes. However, this led to hepatocyte aggregates in distal portal branches, sinusoids, and central veins resulting in severe portal hypertension and hepatic necrosis. (Alwahsh et al. 2018)

The advantages of hepatocyte transplantation are that the procedure is simple compared to whole organ transplantation and one single donor liver can be used for several patients. At least 200–300 g of hepatocytes are needed to effectively sustain life; in theory, these could be transplanted over multiple sessions to reduce complications. The main disadvantage of using implanted hepatocytes is that there is a delay between implantation and the onset of function, which can take 48–72 h after transplantation. Therefore, this technique is not suitable for patients with acute hepatic failure. Furthermore, hepatocyte transplantation requires immunosuppression to prevent rejection, which presents an increased risk of infection in compromised patients. (Attia Atta 2013; Alwahsh et al. 2018; Cardoso et al. 2018)

## Liver bioengineering

Bioengineering approaches are expected to increase the rate of cell engraftment, resulting in increased liver function and stem cell differentiation into acceptable liver tissue. Tissue engineering has become an important technology for liver cell and tissue replacement. Tissue engineering is composed of 3 main components, cells, scaffolding, and signals, such as growth factors. Three-dimensional (3D) culture systems are often used for bioengineering because stem cells can proliferate and differentiate better in 3D culture systems, which resemble the in vivo environment more than 2D culture systems. (Li et al. 2013; Alwahsh et al. 2018)

Artificial livers are being developed to either bridge the patient to transplantation or temporarily support the failing organ until it can regenerate. For the development of the artificial liver, two main approaches are being used: non-biological and hybrid biological-artificial support. The latter uses hepatic cells that are contained within a scaffolding framework. (Court et al. 2003; Pless 2007)

In liver failure, water-soluble toxins (e.g., ammonia and mercaptans) and albumin-bound toxins (e.g., bilirubin, bile acids, aromatic amino acids, and fatty acids) may accumulate and cause encephalopathy and dysfunction in other organs. Detoxification and regulation of liver function can be addressed by artificial devices similar to dialysis (artificial systems, detoxification devices). However, the synthetic function of the liver can only be provided by living cells. To apply these cells safely and conveniently, bioartificial liver support devices are being developed. (Court et al. 2003; Pless 2007)

## Artificial Liver support

Other alternative therapies to replace liver transplantation include plasmapheresis, hemodiafiltration, and artificial livers.(Pless 2007) Disadvantages of these methods include limited availability (in advance medical center facilities only), high cost, anaphylaxis reaction, development-deposition of immune-complexes leading to chronic rejection, and restricted functionality (can only replace 1 or 2 liver functions in the absence of hepatocytes).

Cell-free artificial systems focus on the processes of adsorption and filtration. This approach is based on the assumption that the removal of toxins from the patient’s plasma will improve the clinical state of the patient. Cell-free artificial systems include hemodialysis, hemoperfusion techniques, plasma exchange, and molecular adsorbents recycling systems (MARS). (van de Kerkhove et al. 2004; Pless 2007)

Hemodialysis, a common treatment for renal failure, is also used to treat patients with liver failure to remove water-soluble toxins. (van de Kerkhove et al. 2004) In 1956, Kiley et al. reported the use of hemodialysis in five patients with hepatic encephalopathy. Four patients showed improvements in conscious levels. However, no improvement in long-term survival was observed. (Kiley et al. 1956)

Hemoperfusion using charcoal was first introduced in the 1960s and was found to be effective in removing large molecules in the 500–5000 molecular weight range. In 1976, filtration techniques were further developed with the use of membranes, such as polyacrylonitrile (PAN), which remove higher molecular weight substances. One side effect of these filtration techniques is the incompatibility between blood and the extracorporeal circuit. This results in complement and platelet activation, leukopenia, and the removal of coagulation factors, with the inherent risks of a systemic inflammatory response and catastrophic hemorrhage in patients with pre-existing coagulation defects. Some hormones and growth factors may be unintentionally removed in the process, including hepatocyte growth factor, which has crucial roles in liver regeneration. (van de Kerkhove et al. 2004; Pless 2007)

The cellular components of the blood are separated from the plasma using a plasma filter in plasma exchange/plasmapheresis. Plasma is then replaced by either fresh frozen plasma, albumin solution, or other substitutes. Certain toxins present in the plasma are removed by this process. However, this method requires a large plasma stock and bears the risk of infections. Although biochemical and clinical conditions are improved and toxins are removed by the methods listed above, no substantial survival benefits were observed in patients. (Court et al. 2003; van de Kerkhove et al. 2004)

The MARS is a commercially available system used to filter out albumin-bound toxic metabolites, which eventually lead to encephalopathy and multi-organ failure. The MARS uses albumin-enriched dialysate combined with a charcoal filter and an ion exchange resin. This treatment is capable of removing many protein-bound toxins, including bile acids, endogenous benzodiazepines, mercaptans, and middle- and short-chain fatty acids. Water-soluble substances, such as ammonia, are also filtered out. This system utilizes existing renal dialysis machinery in conjunction with a specially designed device containing a closed-loop albumin circuit. (Court et al. 2003; van de Kerkhove et al. 2004; Pless 2007)

MARS is frequently applied to patients with liver failure. Several single-center experiences and nonrandomized trials have been published. In the first randomized, controlled trial, thirteen patients with cirrhosis were divided into two groups: a control group (n = 5) receiving standard medical treatment and hemodiafiltration (HDF) and a treatment group (n = 8) that incorporated MARS into the standard treatment. MARS treatment was applied 1–10 times for 6–8 h. Creatinine and bilirubin levels decreased significantly while serum sodium levels and prothrombin activity increased significantly in the MARS group. MARS treatment also resulted in significantly prolonged survivals (25 days in the MARS group compared to 4 days in the control group). (Court et al. 2003; van de Kerkhove et al. 2004)

In summary, non-biologic liver support therapies may be beneficial for short-term liver support in moderately affected patients with acute liver failure. However, the nonspecific removal of compounds and the lack of capacity to synthesize liver-specific proteins and other hepatotrophic factors limits their effectiveness. The success of OLT has demonstrated the importance of not only detoxification, but also metabolic functions in patient outcomes. Because these functions can be carried out by hepatocytes, biologic liver support systems are expected to be better at treating ESLD. (Court et al. 2003; van de Kerkhove et al. 2004; Pless 2007)

## Extracorporeal whole liver support therapy

### Xenogenic extracorporeal whole liver perfusion

In 1965, Eiseman reported on extracorporeal pig liver perfusion for the treatment of terminal hepatic encephalopathy. While the patients’ encephalopathy improved, survival rates did not. (Eiseman et al. 1966) Most xenogenic extracorporeal liver perfusions use porcine livers, and this method has been shown to improve neurological outcomes. However, most reports consist of single case applications. Therefore, a definitive conclusion regarding the efficacy of this treatment has not been reached yet. A review of the literature in this field shows no additional survival benefits of porcine extracorporeal liver perfusion over conventional medical therapy for acute liver failure, except when used as a bridge to liver transplantation. In addition, possible transmission of diseases and the activation of xenoantibodies are of concern. There are isolated reports of xenogenic extracorporeal liver perfusions, in which antibodies developed after repeated treatments. However, subsequent perfusions were performed without any detrimental effects. (Court et al. 2003)

### Cross circulation

In 1959, Kimoto investigated cross dialysis between humans and dogs. (Kimoto et al. 1959; Court et al. 2003) Waste products from the patient were metabolized by the canine liver and run through a cation exchange filter. The patient’s clinical condition improved although he died on the 7th day of treatment due to fluid overload and cardiac failure. The patient’s nitrogenous waste products were dramatically reduced and no anti-dog antibodies were detected in the patient’s serum. This suggests that xenogenic hepatocytes could be used without detectable immune activation, if a semipermeable membrane separated the circuits.

## Bioartificial Liver

Bioartificial systems were developed to perform part of the synthetic and regulatory functions of the liver and detoxify patient plasma. As the name implies, bioartificial systems combine liver cells, the biological component, with artificial components, including plastic cartridges with semipermeable membranes. Sources of liver cells include primary cells of either human or xenogeneic origin, cell lines (tumor cell lines or immortalized cell lines), and developing expandable progenitor cell populations. (van de Kerkhove et al. 2004) Primary human cells are biocompatible. Primary cells can be isolated from donor organs that were rejected for transplantation. However, the logistics of receiving human organs and isolating cells are too complicated for large clinical studies. Xenogeneic cells, usually of porcine origin, are more readily available; however, risk of infections and metabolic compatibility are concerning. Most currently available liver cell lines display only a fraction of the metabolic activity of primary human liver cells. Thus, a very large cell mass would need to be applied to achieve therapeutic success. Furthermore, although cells are separated from the patient’s blood by capillary membranes and additional filters, the risk of metastasis formation cannot be excluded. Considering the disadvantages of these cell sources, the ideal cell source is human progenitor cells. However, thus far, investigators have been unable to sufficiently expand and differentiate liver progenitor cells in culture. (van de Kerkhove et al. 2004)

Bioartificial liver support is accomplished using extracorporeal bioartificial liver (BAL) support devices, which combine hepatocytes with plastic cartridges and semipermeable membrane. The most popular systems involve the culture of hepatocytes on the surface of semipermeable capillary hollow fiber membranes within a rigid housing. Nutrient medium is then circulated and communication occurs between the extraluminal and intraluminal compartments through pores in the fiber surfaces. After the hepatocytes have attached and formed an aggregation of liver tissue, the capillary membranes are perfused with the patients’ blood or plasma. Liver cells aggregate via microcarriers on the extraluminal surface or liver cells are trapped within a gel biomatrix within the intraluminal space. (Court et al. 2003)

The following is a list of commercially available BAL support devices. The HepatAssist 2000 (Circe Biomedical, Lexington, MA, USA) is a hollow fiber bioreactor with a cellulose-coated activated charcoal column to remove inorganic toxins and an oxygenator to oxygenate the hepatocytes. The ELAD (VitaGen, CA, USA) is a hollow fiber bioreactor, which uses immortalized human liver cells (C3A) inoculated into the extra-capillary space. The BLSS system (Excorp Medical, Oakdale, MN, USA) is a hollow fiber design and uses primary porcine hepatocytes (cell mass 70–100 g) suspended in collagen medium, which are inoculated into the extra-capillary space. The Modular Extracorporeal Liver Support (MELS) (Charite Virchow Clinic, Berlin, Germany) is a hollow fiber system that uses human hepatocytes from livers deemed unsuitable for transplantation. The MELS combines a CellModule or bioreactor containing hepatocytes with a DetoxModule for albumin dialysis to remove albumin-bound toxins and a DialysisModule for continuous venovenous hemofiltration. The LIVERX2000 system (Algenix/University of Minnesota, MN, USA) is another hollow fiber design, which uses porcine hepatocytes, suspended in colloid solution and injected into the intraluminal space. The AMC-BAL system (Hep-Art Medical Devices B.V, Amsterdam, The Netherlands) uses approximately 200 g of primary porcine hepatocyte aggregates that are immobilized on non-woven polyethylene sheets and rolled separately around each of the polypropylene hollow fibers. (Court et al. 2003)

Several other liver support devices, which avoid the use of hollow fibers, are currently under investigation. These systems utilize woven membranes as scaffolding. Hepatocytes are injected and trapped within the microfibers or encapsulated spheroids of hepatocytes are used. Several new devices are undergoing clinical trials. Problems related to the development of new hybrid BALs include the maintenance of hepatocyte viability and function at the high cell density required for clinical application, the arrangement of the membrane type and structure, the volume of liver tissue required to support a failing liver adequately, and the type of hepatocytes used. In most BAL support devices, the liver cells are separated from the patient’s blood or plasma by at least one membrane. This provides an immunological barrier, but also limits the exchange of substances and, therefore, potentially reduces the effectiveness of the system. Furthermore, the blood/plasma flow is limited to 100–300 mL/min, whereas the blood flow in a normal human liver is about 1500 mL/min, diminishing the maximum clearance. (Court et al. 2003; van de Kerkhove et al. 2004)

In a recent Cochrane Review, trials of artificial and BAL support devices either compared to standard medical treatment (483 patients) or compared to other support systems (105 patients) were summarized. The authors found no general effect on survival in acute liver failure (ALF), but a slight effect in acute on chronic liver failure (AoCLF). They suggest further randomized multicenter studies with larger case numbers. (Liu et al. 2004) A similar conclusion was reached by another systematic review surveying 353 patients with ALF and 130 patients with AoCLF. (Kjaergard et al. 2003)

## Liver organoids

The word organoid per definition is like an organ. An organoid is a miniature version of an organ produced in vitro in three dimensions, which shows a true microanatomy. Organoids, which usually grow from embryonic stem cells or induced pluripotent stem cells, form a three-dimensional culture with the ability to differentiate and self-renew. (McColl 2017) The purpose of organoids is to mimic relevant organ functions and processes from molecular, cellular, tissue, and even whole organ levels using a 3D culture system. (Yin et al. 2016) The characteristics of bioengineered liver organoids are similar to livers, including the cell environment, gene expression, and biological behavior, and these characteristics are influenced by the extracellular matrix. (Li et al. 2013) Stem cell-derived organoids are 3D human microtissues generated in vitro. Liver organoids are expected to recapitulate multiple aspects of the development of a model organ, leading to bioengineered functional liver tissue with the ability to resolve liver diseases, especially ESLD. (Li et al. 2013; Alwahsh et al. 2018)

The theoretical basis of organoid development begins with observations of different species, especially sponges. If destroyed, sponges can compile themselves into new sponges. This observation eventually led to the differential adhesion hypothesis by Malcolm Steinberg. This hypothesis states that differences in cell adhesion intensity play a role in spatial interactions between different cells. Changes in intercellular adherence cause changes in tissue surface tension that affect cell segregation. Cells will reorganize based on surface adhesion differences, to minimize free energy. Cells will move closer to other cells with similar adhesion strength to maximize the bond between cells and produce a more thermodynamically stable structure. When cells with similar surface adhesion bond together, the produced energy due to the existence of these bonds in a system increases and interfacial free energy decreases so that the system will be more thermodynamically stable. This hypothesis is thought to explain cellular movement during morphogenesis, where cells or groups of cells migrate from the initial location to the correct anatomical region. (Steinberg 1996; Foty & Steinberg 2005; Steinberg 2007; Pawlizak et al. 2015)

Human liver organoids are a crucial part of new tissue engineering and therapeutic approaches using stem cells due to the many advantages of organoids. First, liver cell- and MSC-derived organoids have a large proliferative capacity, which can produce millions of cells from a single stem cell in about two months. Second, liver organoids are genetically stable. An analysis of karyotype from liver organoids showed the normal number of chromosomes after several months in culture. Whole-genome sequencing demonstrated that the genomes of liver organoids are quite stable. Third, human liver organoid cells are bi-potent, meaning they can proliferate into hepatocytes and cholangiocytes in response to modifications in the microenvironment. (Ramachandran et al. 2015)

The selection of materials for scaffolding, the cell source, and the culture method are important aspects of developing 3D cultures. The techniques used to culture cells in 3D structures are broadly divided based on whether scaffolds are used. The 3D cultures scaffolds include polymeric hard scaffolds, biological scaffolds, and micropatterned surface microplates, whereas 3D cultures without scaffolds use dropping microplates, spheroid microplates containing ultra-low attachment coating, and microfluidic 3D cell culture. (Larson 2015)

The main two approaches to produce 3D liver organoids are sandwich cultures and spheroid formations. In the sandwich culture method, hepatocyte progenitor cells are implanted layer by layer between collagen or MatrigelTM to resemble liver morphology in vivo and maintain hepatocyte polarity. In spheroid formations, spheroids are formed by hepatocyte progenitor cells cultured alongside stromal cells in a collagen layer. Spheroid formations more closely resemble the physiology of hepatocytes in vivo; cells form tight junctions and become polarized, resulting in improved protein expression and molecular activity. Spheroid formations are also more sensitive to pharmacotoxic components. (Hynds and Giangerco 2013)

The selection of extracellular matrices and bioreactors for 3D culture is also important. The extracellular matrix is often extracted from basement membrane or collagen. MatrigelTM, which is rich in laminin, collagen, growth factors, and enzymes, is often used for the basement membrane. Bioreactors are important for controlling the microenvironment, including temperature, pH, medium flow rate, oxygen, nutrient supply, and metabolic output. (Antoni et al. 2015)

The process of developing 3D cultures of liver organoid is complex. After determining what cells are suitable, the appropriate medium for the formation of organoids needs to be determined, including the extracellular matrix and other microenvironment components. Cells are usually cultured at 37 °C, 5% CO_2_, and 95% humidity. Cells are cultured until they reach 70–80% confluent. Medium and bioreactors are prepared and extracellular matrix components, such as Matrigel™, are added. Incubation is continued so that polymerization can occur and cells are suspended and re-incubated to form organoids. (Ramachandran et al. 2015)

The cell source for liver organoids can be determined by looking at human liver development starting from the endodermal epithelium at the 3rd week of fetal development. Liver buds consist of cells that proliferate and penetrate the septum transversum at the mesodermal plate. These cells develop into liver parenchyma, which consists of hepatoblasts that have the potential to form hepatocytes or cholangiocytes, upon stimulation by fibroblast growth factor (FGF2) or bone morphogenetic proteins. Later, hepatoblasts are regulated by hepatocyte nuclear transcription factors (HND3 and 4) that induce differentiation into hepatocytes and cholangiocytes. The non-parenchymal cells come from a mesenchymal origin. Therefore, the liver organoid needs stem cells that can differentiate into hepatoblasts and other supporting cells that can develop into the sinusoidal and endothelial components. (Zeilinger et al. 2016)

Sources of human liver cells for organoid culture include primary human hepatocytes (PHH), liver cell lines, and stem cells. PHH are the gold standard for in vitro liver models; these cells possess the functions and metabolism of human liver cells. Unfortunately, PHH are difficult to produce in large numbers and are difficult to isolate on a large scale. Liver cell lines from hepatomas or genetically manipulated cells are alternatives to PHH. Liver cell lines are easy to produce on a large scale, but the metabolism of cell lines is often impaired. Stem cells are preferred due to their proliferation and differentiation characteristics. Two types of stem cells are used for liver organoid development, pluripotent stem cells, which originate from embryonic stem cells (ESC) and induced pluripotent stem cells (iPSCs), and adult stem cells (ASC), which originate from liver resident stem cells. (Handa et al. 2014; Willemse et al. 2017)

ASC consist of liver stem cells, small hepatocytes, and progenitor cells. Cytokines, growth factors, hormones, and extracellular matrix proteins affect adult liver stem cells. Mesenchymal stem cells are ASC that can differentiate into cells resembling hepatocytes. Mesenchymal stem cells come from various tissues, such as bone marrow, fat tissue, umbilical cord tissue, or blood. Differentiation can be increased by adding insulin growth factor-1 to the culture medium or by adding inhibitory molecules, such as Rac-1, to accelerate the mesenchymal-to-epithelial transition. (Hindley et al. 2016; Dutta et al. 2017)

Stem cells can be induced to mimic ESC with the capability of differentiating into three germinal layers, ectoderm, mesoderm, and endoderm. These stem cells can then be induced to organize into liver organoids with appropriate differentiation factors. Human-induced pluripotent stem cells (hiPSCs) can be differentiated to produce components similar to native liver cells. One such differentiation method consisted of adding different growth factors at every step of differentiation. For the development of endodermal cells, activin A and Wnt3 are added, in addition to FGF2 and BMP4, and for hepatocyte maturation, hepatocyte growth factor and oncostatin M are added. (Zeilinger et al. 2016)

Liver organoids can also be developed by co-culturing various cells, such as hepatocytes, endothelial cells, and mesenchymal stem cells. If all three components are cultured together in Matrigel™, the cells will organize into organoid structures within 24 h. If the cells are cultured in bioreactors for 10 days, the organoid structures will exhibit liver parenchymal functions, such as cytochrome P450, CYP3A4, CYP2B6, and CYP2C9 activities and mRNA expression. (Ramachandran et al. 2015; Zeilinger et al. 2016)

Liver organoids have some advantages due to their three-dimensional structure, which resembles the in vivo structure and function of the liver. Applications of liver organoids include investigations into organ development and tissue morphogenesis, cell therapy, disease modeling, drug development, toxicology studies, and transplantation, especially for ESLD. (Handa et al. 2014; Hindley et al. 2016; Dutta et al. 2017) In 3D cultures, cells will organize, dynamically interact with each other and the extracellular matrix, and transport nutrients in and out. In this way, 3D cultures resemble in vivo conditions, where cells import nutrition and export waste products into the circulatory system. (Edmondson et al. 2014; Willemse et al. 2017)

For several reasons, 2D cultures do not mimic in vivo conditions as well as 3D cultures. First, liver organoids are more stable and have a longer life span (up to 4 weeks) compared to 2D systems. In 2D cultures, as cells become more confluent they experience contact inhibition. Thus, 2D cultures are not suitable for long-term studies, such as determining long-term drug effects. Second, in 2D culture systems, the cell population becomes homogenous, while in vivo cells are heterogeneous in response to different nutritional supplies. Therefore, a 2D culture system has different metabolic profiles and effects compared to in vivo conditions. Third, relative to 2D culture systems, 3D culture systems have a genetic profile that more closely resembles in vivo cells. Fourth, in 3D systems, cell populations organize differently at different stages or in response to different conditions, including proliferation and resting stages, apoptosis, hypoxia, and necrosis. Fifth, the part of organoids in contact with media will get more and better nutrition and oxygenation, which is similar to the in vivo environment. (Li et al. 2014; Willemse et al. 2017)

There are several limitations to organoids. The scaffolding used in organoids is difficult to extract. Matrices in 3D culture need more components with better composition for optimal construction and these components are costly. Some 3D cultures develop a variety of spheroid sizes causing variability. Lastly, at present, organoids do not develop vascularization. (Edmondson et al. 2014; Willemse et al. 2017)

## Inventions of liver engineered organoids

Although there are many in vitro experiments with organoids, there are very few in vivo experiments. In 2013, Takebe et al. were the first to generate liver organoids from iPSCs. These organoids formed liver buds, which were transplanted into mice to explore organoid function and compatibility. Gancyclovir was administered to immune-deficient (TK-NOG) mice to develop liver failure. The survival of mice that received the liver organoids increased compared to the sham group or mice that received adult hepatocytes or fetal liver cell-derived liver buds. (Takebe et al. 2013)

In 2018, Nie et al. were the first to introduce liver organoid transplantation in mice from a single donor. Cells were obtained from a single donor to limit the different HLA types, a condition that was more applicable to the clinical situation. In this experiment, the liver organoid was a 3D-cell culture complex consisting of endothelial stem cells (ECs), umbilical cord-derived mesenchymal stem cells, and ECs derived from hiPSCs. The complex was cultured and differentiated for several days before the cells displayed hepatic cell lineage. The hepatic cell lineage complex was then transplanted into mice with ALF. The ALF condition was generated by administering 1.5 µg/kg of diphtheria toxin, resulting in the macroscopic and histological appearance of hepatocellular damage, necrosis, and steatosis, as well as increased liver enzymes (AST > 10.000 IU/L and ALT > 15.000 IU/L). After transplantation of the hepatic cell lineage complex into the renal subcapsular space, 70% of mice recovered, AST and ALT levels were decreased, human albumin was detected by ELISA, and the hepatic cord structure, proving the presence of the liver organoid, was histologically observed. (Nie et al. 2018) This study gives support for the use of liver organoids in clinical settings. However, to our knowledge, there are no clinical trials using liver organoids yet. Clinical trials using liver organoids is a promising future research endeavor.

BALs and liver organoids have their own advantages and limitations. However, the effort to combine both methods is promising. The prospect of liver organoids that utilize stem cells instead of the ‘impractical’ adult hepatocytes could be applied to the complex problem of developing a BAL. Some recent studies have explored the benefit of differentiating mature hepatocytes from stem cells inside a bioreactor. This approach has also been considered for the development of a BAL. Huang et al. and Shi et al. have both used human-induced Hepatocytes (hiHeps) in mice and pigs with liver failure. The promising results include decreased AST and ALT levels, detection of human albumin by ELISA, and longer survival rates for the mice and pigs. (Huang et al. 2014; Shi et al. 2016) Moreover, Huang et al. discovered that BAL treatment decreased the infiltration of inflammatory cells into liver tissue by down-regulating the production of cytokines, such as TNFα and IFNγ. (Huang et al. 2014)

## Future perspectives

**Fig. 1:**
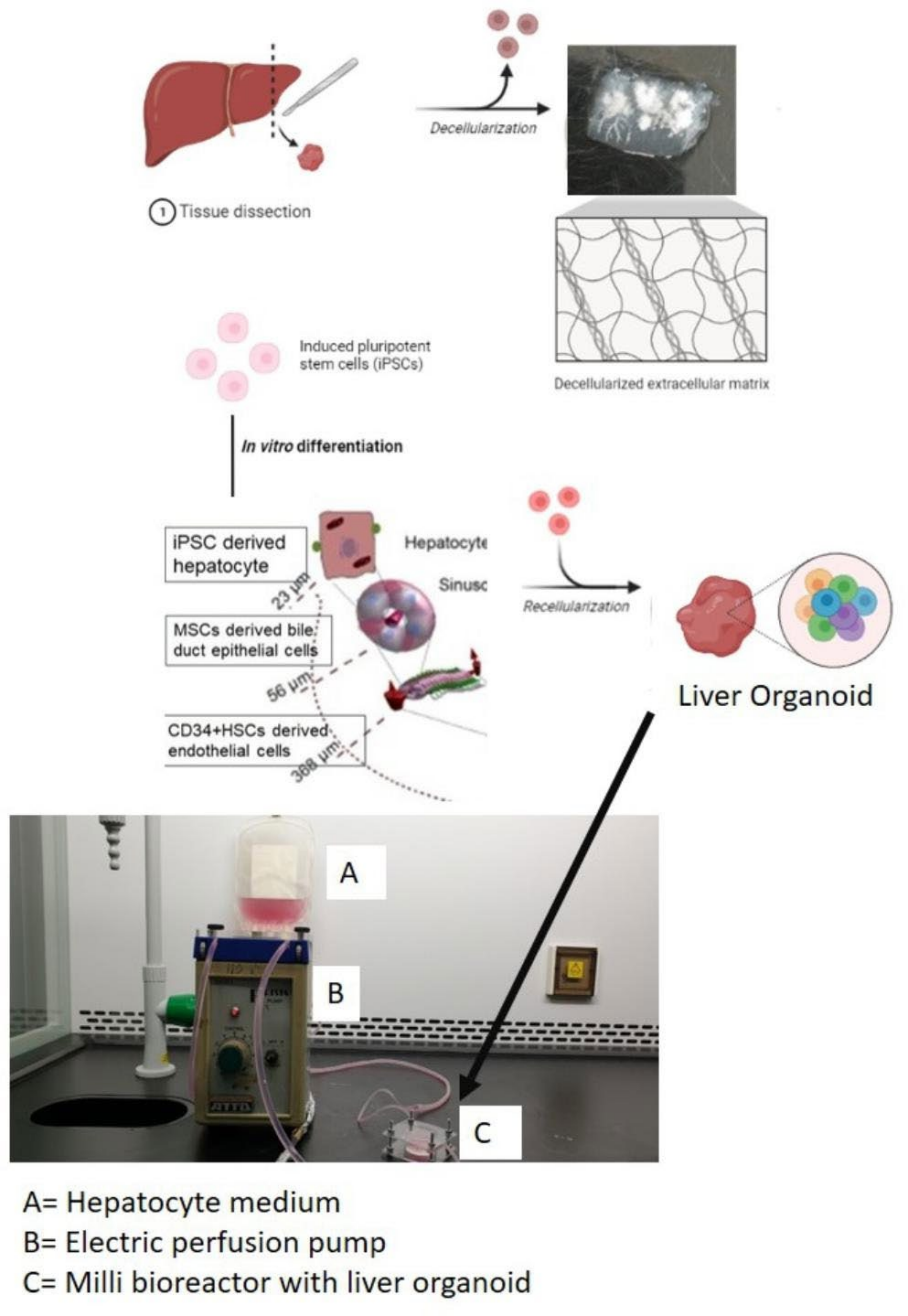
SHiNTA (Stem cell Hepatic INTuitive Apparatus) initial prototype in Faculty of Medicine Universitas Indonesia uses iPSCs derived hepatocytes in decellularized liver scaffold. Liver organoid from iPSCs derived hepatocytes, MSCs derived bile duct epithelial cells, and CD34 + HSC derived endothelial cells in decellularized liver scaffold will be the complete SHiNTA prototype. This figure is made with combination of education material from biorender.com, photograph of decellularised liver scaffold which was published in AIP Conference Proceedings (Antarianto et al. 2019) and of SHiNTA prototype which was registered Indonesian patent (Akhmadu et al. 2021)

Engineered liver organoids will progress into artificial liver prototypes with the end target of a low-end ambulatory liver support unit. Inventing engineered liver organoids requires the formation of liver organoids inside a portable bioreactor. Liver organoids can be obtained from a combination of cellular components in 3D co-cultures, including iPSCs derived hepatocytes, MSCs derived bile duct epithelial cells, and CD34 + HSC derived endothelial cells. Infusing or seeding these organoids into a milli bioreactor (Fig. 1) will produce engineered liver organoids for low-end artificial liver prototypes. A portable small bioreactor (milli bioreactor) design with inlets and outlets enables the replacement of culture medium or removal of metabolic waste and monitoring. This apparatus is expected to function as a liver replacement for terminal liver failure patients in peripheral or remote areas that don’t have access to liver transplantation. At the moment, the prototype in Faculty of Medicine Universitas Indonesia uses iPSCs derived hepatocytes in decellularized liver scaffold.
